# Resilience in the General Population: Standardization of the Resilience Scale (RS-11)

**DOI:** 10.1371/journal.pone.0140322

**Published:** 2015-11-02

**Authors:** Rüya-Daniela Kocalevent, Markus Zenger, Ines Heinen, Sarah Dwinger, Oliver Decker, Elmar Brähler

**Affiliations:** 1 Institute and Policlinic for Medical Psychology, University Medical Center Hamburg-Eppendorf, Hamburg, Germany; 2 Department of Medical Psychology and Medical Sociology, University of Leipzig, Leipzig, Germany; 3 Faculty of Applied Human Studies, University of Applied Sciences Magdeburg and Stendal, Stendal, Germany; 4 Department of Psychosomatic Medicine and Psychotherapy, Universal Medical Center Mainz, Mainz, Rhinland-Palatinate, Germany; University of Vienna, School of Psychology, AUSTRIA

## Abstract

**Background:**

The objectives of the study were to generate normative data for the RS-11 for different age groups for men and women and to further investigate the construct validity and factor structure in the general population.

**Methods:**

Nationally representative face-to face household surveys were conducted in Germany in 2006 (n = 5,036).

**Results:**

Normative data for the RS-11 were generated for men and women (53.7% female) and different age levels (mean age (SD) of 48.4 (18.0) years). Men had significantly higher mean scores compared with women (60.0 [SD = 10.2] vs. 59.3 [SD = 11.0]). Results of CFA supported a one-factor model of resilience. Self-esteem (standardized β = .50) and life satisfaction (standardized β =.20) were associated with resilience.

**Conclusions:**

The normative data provide a framework for the interpretation and comparisons of resilience with other populations. Results demonstrate a special importance of self-esteem in the understanding of resilience.

## Introduction

Most definitions of resilience emphasize two elements as crucial [1–3]. First, an input perspective: the exposure to risk and adverse circumstances, which can vary from moderate to extreme risks environments. The second element of a resilience definition is in respect to an outcome perspective, studying whether coping mechanisms lead to outcomes within or above the expected range. This should be assessed by comparing the outcome to a context specific reference group (e.g. same age group, social and cultural context, etc.) [4]. According to Rutter, the concept of resilience has to be considered on the basis of evidence of risk and protection [5]. Particularly during the last two decades, there has been a marked tendency for researchers, clinicians, and policy makers to shift their focus from risk to resilience, whereby resilience represents the interaction between risk factors (vulnerability) and resources (protection) [6].

Evidence suggests that, amongst others, positive emotions are an important source of individual or personal resilience [7]. For example, the tendency to use pleasant daily life experiences to boost positive emotions (positive affect reactivity) is associated with increased resilience against depressive symptoms in the future [8,9]. Increases in positive emotion (rather than decreases in negative emotion) might also predict recovery from depression [10]. Moreover, the experience of positive emotions also attenuated the degree to which genetic vulnerability for depression was expressed as a negative mood bias [11]. Besides depression, life satisfaction, as one positive indicator of mental health, is also strongly associated with resilience both in men and in women in the general population [12,13]. With regard to stability over the course of life, studies suggest that levels of resilience are relatively independent of age with a slight decrease in older age [14,15]. One model of resilience has been proposed by Haase (2004), taking into account protective factors (e.g. social support) and risk factors (e.g. distress) [16]. According to the researchers, one main outcome factor depicted by the model includes self-esteem, and a second outcome includes quality of life, defined as a general sense of well-being.

Research on self-esteem has its origin in social psychology [17]. Rosenberg defines self-esteem as a component of the self-concept, an individual’s positive or negative thoughts and feelings about her or his worth and importance. Self-esteem is considered a stable sense of worth or worthiness. Rosenberg’s thoughts on self-esteem arise from the idea that people’s attitudes towards themselves resemble their attitudes towards other objects. He claims that people’s attitudes have a very strong effect on how they see themselves. Empirical findings indicate that self-esteem and resilience are intricately linked, with associations ranging from *r* =.21 to *r* =.51 [18–20]. Self-esteem was inconsistently associated with depression/anxiety symptoms, yet higher resilience scores were linked with lower depression/anxiety symptoms [21–24] [25,26]. In some studies, the constructs of self-esteem and resilience are used synonymously [16,27,28]. In sum, self-esteem is either described synonymously as an indicator of resilience, or as a general indicator of psychological adjustment. The definition emphasizes the aspect of resilience is a process rather than a static concept or an individual characteristic, as for example self-esteem [6]. There is an interaction and adaptation process that occurs after the individual is exposed to an adversity. As Rutter 1987 emphasizes, such a dynamic perspective helps to avoid misunderstandings of the concept of resilience as a fixed personal characteristic: ‘resilience cannot be seen as fixed attributes of the individual. If circumstances change, the risk alters’ [29].

Identifying and measuring individual or personal resilience contributes to our understanding of stress resistance and successful adaptation, including physical health [28,30,31] and mental health [32–34] in different health care settings and health promotion in communities. Recently the routine assessment of resilience has become more prominent in mental health research [35–38]. Levels of resilience vary between people [32]. To assess the level of resilience, it is necessary to relate the individual score to a reference group. Normative scores can be used to compare scores from different settings with those from the general population as well as separately for sex and age groups.

Ahern et al. (2006) indicated in their review that the Resilience Scale may be the best to use with adolescent population [39]. Other scales were lacking evidence for their use at this time largely due to a lack of research applications. Multiple applications of the Resilience Scale were available in both sexes, multiple ages, and ethnic groups with good reliability and validity [39].

The Resilience Scale was first published in 1993, comprising 50 items and two factors: “personal competence” and “acceptance of self and life” [40]. Twelve completed studies, in a variety of settings and with diverse samples using the resilience scale, were reviewed by one of the original authors in 2009 [41]. Lack of resilience was significantly related to hopelessness, loneliness, and life-threatening behaviours. Hopelessness and social connectedness explained 50% of the variance in resilience. Resilience and life satisfaction were the strongest predictors of well-being. Further contributions to the construct validity of the resilience scale yielded negative associations with depression, anxiety, and stress and positive correlations with physical health, life satisfaction, and health promoting activities [39,42,43]. Standard total scores, without age subgroups within sex, were reported by Schuhmacher et al. (2005) for shorter versions of the original Resilience scale, the RS-25 and RS-11 [44]. Schuhmacher et al. reported the RS-11 to be unidimensional, with 9 items from the original “personal competence” factor and 2 items from the original second factor “acceptance of self and life”.

The main aims of the present study were test psychometric properties and dimensionality of the RS-11, to test for differences related to sociodemographic variables and to provide normative data for the RS-11, a shorter version of the original resilience scale [14], for a population sample of different age groups and for both men and women. In addition, we address the relations of resilience with self-esteem and life satisfaction, as well as with depression and anxiety, in order to provide further evidence for the construct validity. Our focus on self-esteem was guided by research from a risk and resilience perspective, which has emphasized self-esteem as a protective personal resource when individuals are faced with adversity [45–47].

## Methods

### Study sample

A nationwide survey, representative of the German general population, was conducted with the assistance of an institute specialized for demographic research (USUMA, Berlin) according to the German law of data protection (§30a BDSG, German law of protection of data privacy) and with written consent and in accordance with the guidelines in the Declaration of Helsinki. The ethics committee of the University of Leipzig approved the study. All adult participants provided their written informed consent to participate in this study. Also, written informed consent from the next of kin, caretakers, or guardians on behalf of the minors/children enrolled in the study was obtained. These consent procedures were approved by the ethics committee. The basic population for the data collection is made up of the German population aged at least 14 years and living in private households in 2006 (N = 5,036). Age, sex, and educational level were the major criteria for representativeness according to the register of the Federal Elections. Two callbacks had to be without success before an address was considered a failure. The sampling procedure consisted of sample points, household, and persons in the last stage. Target households within the sample points were determined using the random-route procedure: choosing sample point areas within Germany, randomly choosing households within these areas, and randomly choosing target persons within these households.

Within this larger survey, the study participants were interviewed using a structured self-report questionnaire including the following instruments.

### Instruments

#### Resilience (Resilience Scale; RS-11)

The purpose of the Resilience Scale is to identify the degree of individual resilience, “…considered a positive personality characteristic that enhances individual adaptation” [40,48]. Here resilience is measured by the 11-item short form (RS-11) validiated by Schumacher et al. [14]. Resilience, in the brief 11-item version, is conceptualized as a protective personality factor that is associated with a healthy development and psychosocial stress-resistance, using a 7-point Likert scale “from ‘1’ = *strongly disagree* to ‘7’ = *strongly agree*”. The RS-11, conceptualized as a unidimensional scale, has shown to be a reliable and valid instrument that allows an economic assessment of resilience in a community sample of N = 2,031 [14,49].

#### Depression (Patient-Health Questionnaire; PHQ-2)

The PHQ-2 includes the first 2 items of the PHQ-9 [50,51]. The items correspond to the first two DSM-IV Diagnostic Criterion A symptoms for major depressive disorder (“Feeling down, depressed, or hopeless”; “Little interest or pleasure in doing things”) [52].

Response options are “not at all”, “several days”, “more than half the days”, and “nearly every day”, scored as 0, 1, 2, and 3, respectively, using a 4-point Likert scale.

The validity of this two-item depression screener is well documented [53]. Cronbach α in the present study was .78

#### Anxiety (Generalized Anxiety Disorder; GAD-7)

The GAD-7, which was designed to identify probable cases of generalized anxiety disorder and to assess symptom severity, evidenced high reliability and validity in primary care patients and in the general population [54,55] The GAD-7 items describe the most prominent diagnostic features of the DSM-IV diagnostic criteria A, B, and C for generalized anxiety disorder [52].

Response options are “not at all”, “several days”, “more than half the days”, and “nearly every day”, scored as 0, 1, 2, and 3, respectively, using a 4-point Likert scale.

Internal consistency in this study was α =.89 (Löwe et al., 2008).

#### Self-esteem (Self-Esteem Scale; RSES)

The German Adaptation of Rosenberg’s Self-Esteem scale (RSES) was administered [56]. The RSES is composed of five positively (e.g. “I am satisfied with myself.”) and five negatively worded items (e.g. “At times, I think I am not good at all.”) with four response categories “from ‘0’ = *strongly agree* to ‘3’ = *strongly disagree*”, using a 4-point Likert scale. Psychometric properties of the scale are well documented, including Cronbach α =.88 [57].

#### Life satisfaction (Life satisfaction scale; FLZ^M^)

Life satisfaction reflects aspects of a general sense of well-being. The questions on Life Satisfaction (FLZ^M^) are a multi-dimensional self-report measure of general life satisfaction and satisfaction with health with established international normative data [58]. The general domains cover friends, leisure time activities/hobbies, general health, income, profession, housing/living conditions, family life, and partnership/sexuality. Respondents weight their satisfaction with each of the eight domains of daily life in relation to the subjective importance of the domain. In the first step, respondents rate the subjective importance of each dimension on a scale “from ‘1’ = *not important* to ‘5’ = *extremely important*”. Then they rate the present satisfaction with these dimensions on a scale from ‘1 = dissatisfied’ to ‘5 = very satisfied’, using a 5-point Likert scale. Cronbach α in the present study was .83.

### Data analysis

As measures of the test’s reliability, both Cronbach’s alpha and McDonald’s omega were calculated. The factor structure of the RS-11, using a 7-point Likert scale, was tested with confirmatory factor analysis (CFA), using the maximum likelihood approach, with data being treated as of a continuous scale, according to Beauducel & Herzberg (2006), who indicated that maximum likelihood based fit indices can only be affected by low number of categories (<5) [59]. The model fit of the CFA was tested using the following fit indices: the minimum discrepancy, divided by its degrees of freedom (CMIN/DF); the goodness-of-fit-index (GFI); the normed-fit-index (NFI); the Tucker-Lewis-Index (TLI); the comparative-fit-index (CFI); standardized root mean square residual (SRMR); and the root mean square error of approximation (RMSEA). For a good model fit, the ratio CMIN/DF should be closed to 3 or even smaller [60]. Furthermore, values of GFI, NFI, TLI, and CFI values higher than 0.90 were initially advanced for an indication of an acceptable model fit, but due to ensure that misspecified models are not accepted, a cutoff value of ≥0.95 for CFI and TLI is now preferred (Hooper, Coughlan, Mullen 2008). Values for RMSEA should be <0.10, and SRMR should be 0.05 or smaller [60,61]. Additional analyses were conducted to test the invariance of the model across sex and different age groups using multi-group CFA. This is an important statistical condition before means of different subgroups can be compared with each other [62]. Measurement invariance was tested in three steps using first the configural model (no constraints), followed by a metric invariant model (with equal item loadings), and a scalar invariant model (with equal item loadings and item intercepts across groups) [63]. Since these models are hierarchically nested and increasingly restricted, the models were then compared to each other on the basis of the differences ΔCFI and ΔRMSEA. Values ≤.01 indicate the invariance of the models [64].

For reliability, McDonald’s Ω_w_ of all measures used in this study was assessed as an indicator of construct reliability (Brunner & Süß, 2005). In addition, we investigated group differences for sociodemographic characteristics using χ^2^-test and Kruskal-Wallis-test (p<0.001), respectively. To provide normative data for the RS-11, we generated age subgroups within sex specific percentiles for the total score. Correlation coefficients were corrected for attenuation due to lower estimates of internal consistencies because of the shortness of the scales used in this study (Spearman, 1904). Additionally, independent variables (self-esteem, life satisfaction, depression, anxiety) were entered into multiple regression analyses irrespective of statistical significance based on both pearson correlation coefficients and disattenuated correlation coefficients. Our aim was to check what relationships with the dependent variable (resilience) would look like if the correlations were corrected for attenuation.

The percentiles were calculated according to the following formula [65]: percentile rank = 100* (m + 0.5 k)/N, where m is the number of members of the sample who obtained a score that was lower than the score of interest, k is the number who obtained the score of interest, and N is the overall normative sample size.

Statistical analyses were conducted using SPSS with an α-level of 5% and AMOS 20.

## Results

### Sample characteristics

The survey was carried out by professional interviewers from a demographic consultation company (USUMA, Berlin). Within each wave, a representative sample of the German population aged 14 years or older was approached using 258 sample points. Addresses were selected according to the random route procedure. Of the 8,398 addresses selected, 8,106 proved valid. A total of 5,036 persons agreed to participate, provided verbal informed consent, and completed the study questionnaires. The response rate among those individuals who were asked to participate by the interviewers was 62.1%.

Characteristics of the study sample closely match those of the total German population [66] and the US National Comorbidity Survey Replication [67] on gender (women: 53.7%, 51.7%, and 55.5%, respectively), employment status (unemployed: 5.8%, 7.1%, and 3.9%, respectively), marital status (married: 53.7%, 57.2%, 57.2%), and educational level. In addition, mean age in our study sample was similar to the mean age in the German general population aged 14 years or older (48.4 vs. 46.9 years).

**Fig 1:**
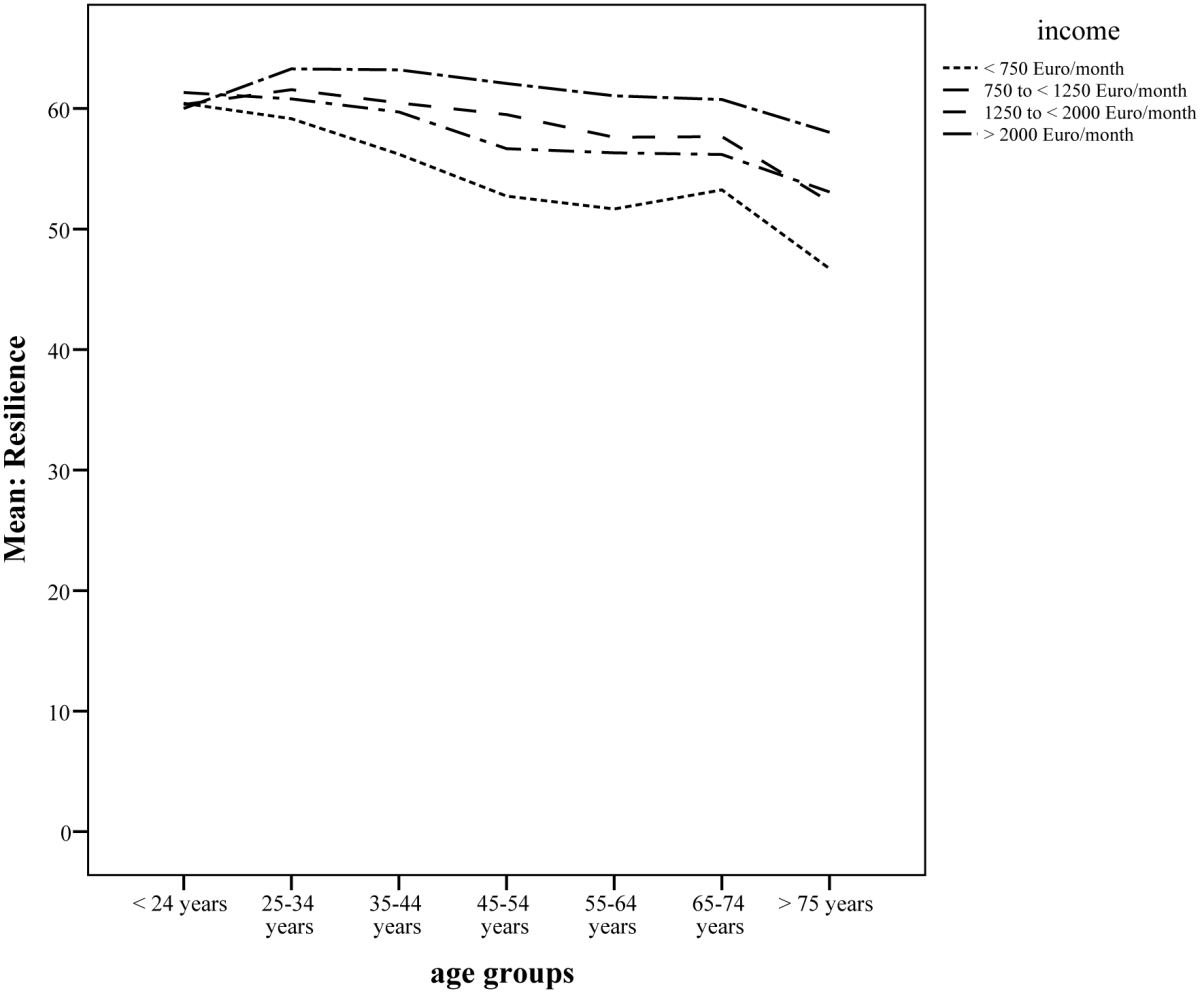
Significant interaction of age groups and monthly income (dependent variable: resilience).

Effect sizes and confidence intervals were calculated according to Hedges & Olkin (1985). In each socioeconomic category the first subgroup was used as a reference group, and in case of more than two subgroups per category, the total sample standard deviation was used to compute effect sizes (instead of a pooled standard deviation) to put values on a comparable metric. There were significant gender, age, marital status, education level, employment status, and income effects in the general population associated with a higher RS-11 score. As noted in Table 1, the calculated effect sizes were small for gender and employment status, and moderate to high for the other sociodemographic groups, with age group >75 years having the largest effect size (*d* = 0.71, CI: 0.573–0.843) for low resilience and the group of net household income ≥ 2500€/month having the largest effect size (*d* = 0.59, CI: 0.499–0.671) for high resilience. A two way ANOVA yielded significant main effects for age groups (F = 17.52; df = 6; p<0.001) as well as for income (F = 33.52; df = 2; p<0.001) and significant interaction of age groups and income (F = 2.525; df = 12; p<0.01). The higher the age, the higher the significance of higher income for the amount of reported resilience (see Fig 1).

**Table 1:** Demographic characteristics of the study sample and associations with RS-11 scores.

	N (%)	RS-11 M (SD)	Cohen’s *d* effect size^1^	Confidence interval
Gender				
Male	2334 (46.3)	60.0 (10.2)		
Female	2702 (53.7)	59.3 (11.0)	*d* = 0.06	.009-.119
Age group, yr.				
14–24	558 (11.1)	60.4 (10.8)		
25–34	684 (13.6)	62.0 (10.3)	*d* = 0.15	.041-.265
35–44	964 (19.1)	61.8 (9.8)	*d* = 0.13	.029-.238
45–54	863 (17.1)	60.3 (10.1)	*d* = 0.00	-.103-.110
55–64	808 (16.0)	58.5 (10.3)	*d* = 0.18	.070-.287
65–74	784 (15.6)	57.8 (10.6)	*d* = 0.24	.132-.350
≥ 75	375 (7.4)	52.8 (11.4)	*d* = 0.71	.573-.843
Cohabitation				
Yes	3014 (59.8)	60.3 (10.3)		
No	2022 (40.2)	58.6 (11.1)	*d* = 0.16	.102-.215
Marital Status				
Married	2702 (53.7)	60.1 (10.3)		
Separated	63 (1.3)	58.4 (8.5)	*d* = 0.15	-.098-.402
Single	1220 (24.2)	60.8 (10.8)	*d* = 0.07	-.001-.134
Divorced	475 (9.4)	59.8 (10.2)	*d* = 0.02	-.076-.119
Widowed	576 (11.4)	54.9 (11.3)	*d* = 0.48	.393-.574
Education				
High School	4094 (81.3)	59.2 (10.7)		
College	384 (7.6)	62.7 (9.7)	*d* = 0.34	.232-.442
University	328 (6.5)	62.7 (9.3)	*d* = 0.33	.217-.442
Currently Student	174 (3.5)	59.8 (10.4)	*d* = 0.06	-.094-.210
None	56 (1.1)	52.6 (11.1)	*d* = 0.61	.347-.875
Unemployment				
Yes	293 (5.8)	55.9 (11.4)		
No	4743 (94.2)	59.8 (10.6)	*d* = 0.38	.257-.494
Net household income				
< 1250 €/month	1071 (21.3)	56.8 (11.4)		
1250-<2500 €/month	2620 (52.0)	59.3 (10.4)	*d* = 0.23	.159-.301
≥ 2500 €/month	1080 (21.4)	63.1 (9.6)	*d* = 0.59	.499-.671

^1^Cohen’s defined effect sizes as follows: “small, d =.2”, “medium, d =.5”, and “large, d =.8”.

### Internal consistency

The parameter of internal consistency (Cronbach’s α) for the RS-11 scale reached the value of α = 0.92, Ω_w_ as an indicator of construct reliability was also .92.

### Construct validity

#### Factor analysis

The unidimensional structure of the RS-11 was tested according to Schuhmacher et al. (2005) using CFA with N = 5,036 participants. Most of the fit indices indicated at least an acceptable model fit (GFI =.928; NFI =.927; SRMR =.038; RMSEA =.097), while two fit indices were somewhat smaller than the recommended cutoff criterion (TLI =.911, CFI =.929). The value of CMIN/DF (CMIN/DF = 48.46) indicated a relevant deviation between the data and the model, but the χ^2^-statistic has often been criticized for its sensitivity to sample size. Thus, in case of large sample sizes, even a small misspecification would lead to a rejection of the model [68]. Therefore, we focused on the fit indices mentioned above, which are more independent of the sample size. To sum up, the unidimensionality of the RS-11 can be confirmed, even if two of six fit indices did not reach the cutoff criterion. Standardized factor loadings ranged between .57 and .80.

In the following section we tested for invariance of the model across gender and age (see Table 2).

**Table 2:** Test for invariance across gender and age using multi-group CFA.

	Chi^2^ (df)	ΔChi^2^	Δp	CMIN/DF	CFI	Δ CFI	RMSEA	Δ RMSEA
***Multigroup analysis—Gender***								
Configural invariance model	2,210.956 (88)			25.125	.928		.069	
Metric invariance model	2,234.311 (98)	23.355	.010	22.799	.927	.001	.066	.003
Scalar invariance model	2,370.967(109)	160.011	<.001	21.752	.923	.004	.064	.002
***Multigroup analysis—Age***								
Configural invariance model	2,604.788 (264)			9.867	.918		.042	
Metric invariance model	2,680.186 (314)	75.398	.012	8.536	.917	.001	.039	.003
Scalar invariance model	3,261.818 (369)	581.632	<.001	8.840	.898	.019	.040	.001
Partial scalar invariance model (without items 5 and 11)	3,005.745 (359)	325.559	<.001	8.373	.907	.010	.038	.001

As shown in Table 2, configural, metric and scalar invariance across both genders could be confirmed, as ΔCFI and ΔRMSEA values were ≤.01. Regarding the invariance across several age groups, configural and metric invariance could be confirmed, but due to ΔCFI =.02, scalar invariance could not. Modification indices indicated that item 5 and item 11 significantly contributed to the lack of fit, and therefore the constraint of equal intercepts was freed for these items (according to [69]). Afterwards, the model was reestimated for partial scalar invariance. As shown in Table 2, partial invariance across all age groups could be confirmed.

Regarding the content of item 5 (“I feel that I can handle many things at a time”) and item 11 (“I have enough energy to do what I have to do”), these items might reflect decreasing capabilities with increasing age. We observed a slight trend of decreasing intercepts with increasing age and the biggest drop down in the oldest age group >70 years (intercepts of item 5 in six age groups: (1) 5.21; (2) 5.34; (3) 5.24; (4) 5.01; (5) 4.66; (6) 4.03; intercepts of item 11: (1) 5.67; (2) 5.76; (3) 5.66; (4) 5.47; (5) 5.34; (6) 4.71).

#### Intercorrelations

Table 3 now presents the entire intercorrelation matrix of the 5 scales. Intercorrelations with resilience were highest for self-esteem (*Pearson correlation coefficient*:59 p <.001; disattenuated correlation coefficient: .66), followed by life satisfaction (*Pearson correlation coefficient*:.41 p < .001; disattenuated correlation coefficient: .47). Disattenuated values are simliar to Pearson correlation coefficients, indicating that the reliability of all scales is not an issue in the interpretation of results.

**Table 3:** Intercorrelations of resilience, life satisfaction, self-esteem, depression, and anxiety (N = 5,036).

	Resilience (RS-11)	Self-esteem (RSES)	Life satisfaction (FLZ^M^)	Depression (PHQ-2)	Anxiety (GAD-7)	Regression to RS-11 β (pearson correlation coefficients)	Regression to RS-11 β(disattenuated correlation Coefficients)
Resilience (RS-11)	1	.59 (CI: .57-.61)	.41 (CI:.39-.44)	-.34 (CI: -.36—.31)	-.29 (CI:-.31—.26)		
Self-esteem (RSES)	.66	1	.42	-.48	-.43	.50	.58
Life satisfaction (FLZ^M^)	.47	.49	1	-.41	-.39	.20	.21
Depression (PHQ-2)	-.40	-.58	-.51	1	.64	-.04	-.02
Anxiety (GAD-7)	-.32	-.49	-.46	.77	1	.04	.08

Note: Pearson correlation coefficients above the diagonal; disattenuated correlation coefficients below the diagonal; all p < .001. CI: Confidence Interval.

Additionally, Table 3 reports on the results of the multiple regressions (a) on the Pearson correlation coefficients and (b) on the disattenuated correlation coefficients. Relationships with the dependent variable (resilience) look alike for correlations corrected for attenuation. The regression analysis showed (R^2^corr =.35, p < .001) that 35% of the variance in the dependent variable, resilience, is accounted for by self-esteem.

Self-esteem (standardized β = .50) and life satisfaction (standardized β =.20) were both associated with resilience.

### Normative data

Table 4 summarizes the normative data for the different age levels and both genders. Percentiles from this table can be used to compare an individual subject’s RS-11 score with those determined from the general population reference group based on age and gender.

**Table 4:** Normative data from the general population for the RS-11.

Total		Men					Women				
	14–92 y N = 5,036	14–92 y n = 2,328	14–25 y n = 335	26–40 y n = 491	41–60 y n = 808	≥ 61 y n = 694	14–92 y n = 2,695	14–25 y n = 299	26–40 y n = 684	41–60 y n = 915	≥ 61 y n = 797
**M**	59.61	59.97	60.78	61.96	60.19	57.91	59.29	60.72	61.52	60.56	55.39
**SD**	10.65	10.20	9.71	9.74	10.15	10.44	11.02	11.78	10.52	10.03	11.25
**Sum Score**	**Percentile**										
11	0.0	0.0	0.0	0.0	0.0	0.0	0.0	0.0	0.0	0.0	0.0
12	0.0	0.0	0.0	0.0	0.0	0.0	0.0	0.0	0.0	0.0	0.0
13	0.0	0.0	0.0	0.0	0.0	0.0	0.0	0.0	0.0	0.1	0.0
14	0.0	0.0	0.0	0.0	0.0	0.0	0.1	0.0	0.2	0.1	0.0
15	0.1	0.0	0.0	0.0	0.0	0.0	0.1	0.0	0.3	0.1	0.0
16	0.1	0.0	0.0	0.0	0.0	0.0	0.2	0.0	0.4	0.1	0.2
17	0.2	0.0	0.0	0.0	0.0	0.1	0.3	0.3	0.4	0.1	0.4
18	0.2	0.0	0.0	0.0	0.0	0.1	0.4	0.7	0.6	0.1	0.4
19	0.2	0.0	0.0	0.0	0.0	0.1	0.4	0.7	0.6	0.1	0.4
20	0.2	0.0	0.0	0.0	0.0	0.1	0.4	1.0	0.6	0.1	0.4
21	0.3	0.0	0.0	0.0	0.0	0.1	0.5	1.3	0.6	0.2	0.4
22	0.3	0.0	0.0	0.0	0.0	0.1	0.6	1.3	0.6	0.3	0.6
23	0.4	0.1	0.3	0.0	0.0	0.3	0.6	1.3	0.6	0.3	0.8
24	0.5	0.3	0.6	0.0	0.3	0.4	0.7	1.7	0.6	0.4	0.8
25	0.6	0.4	0.6	0.0	0.5	0.6	0.8	2.2	0.7	0.4	0.9
26	0.8	0.5	0.6	0.0	0.6	0.7	1.0	2.2	0.7	0.5	1.3
27	0.9	0.6	0.9	0.0	0.6	0.9	1.1	2.2	0.7	0.5	1.5
28	0.9	0.7	0.9	0.0	0.7	1.2	1.1	2.2	0.7	0.5	1.6
29	1.0	0.8	1.2	0.0	0.7	1.3	1.2	2.7	0.7	0.5	1.8
30	1.1	0.9	1.2	0.0	0.9	1.3	1.2	2.7	0.7	0.5	1.9
31	1.2	0.9	1.2	0.0	1.1	1.4	1.4	3.0	0.9	0.7	2.1
32	1.3	1.1	1.2	0.0	1.1	1.7	1.5	3.0	0.9	0.7	2.3
33	1.4	1.1	1.2	0.0	1.2	1.9	1.6	3.3	0.9	0.8	3.6
34	1.6	1.3	1.2	0.0	1.7	2.0	1.8	3.7	0.9	1.0	2.9
35	1.8	1.6	1.2	0.0	2.1	2.4	2.1	4.0	1.0	1.3	3.3
36	2.2	1.8	1.2	0.2	2.4	2.8	2.5	4.4	1.2	1.6	4.1
37	2.6	2.0	1.2	0.4	2.6	3.0	3.0	4.9	1.7	1.9	5.0
38	2.9	2.3	1.6	0.6	2.8	3.3	3.5	5.4	2.1	2.0	5.8
39	3.4	2.7	2.2	1.0	2.3	3.8	4.0	5.4	2.4	2.2	6.8
40	3.9	3.3	2.7	1.5	3.7	4.5	4.5	5.7	2.4	2.6	8.0
41	4.5	3.8	3.1	1.9	4.0	5.3	5.1	6.0	2.9	3.0	9.4
42	5.4	4.5	3.9	2.6	4.6	6.2	6.1	6.9	3.5	3.5	11.4
43	6.6	5.7	5.2	3.7	5.8	7.4	7.3	7.9	4.3	4.3	13.4
44	8.2	7.3	6.6	4.9	7.4	9.4	9.1	8.9	5.9	5.5	16.1
45	10.2	9.0	7.3	6.3	8.9	11.9	11.2	10.2	8.1	7.2	19.1
46	11.8	10.4	7.8	7.6	10.2	14.1	13.0	11.2	9.6	8.7	21.8
47	13.4	11.9	9.0	9.2	11.6	15.8	14.7	12.2	10.5	10.0	24.7
48	15.1	13.7	10.9	10.7	12.9	18.2	16.4	13.4	11.5	11.5	27.4
49	16.8	15.3	12.4	11.6	13.9	21.2	18.0	14.2	12.2	13.2	30.2
50	18.5	16.9	14.0	12.7	15.1	23.6	19.8	15.1	13.5	14.8	32.9
51	20.4	18.8	15.8	14.3	16.8	26.0	21.7	16.4	15.5	16.6	35.2
52	22.4	20.7	17.2	15.9	18.6	28.6	23.9	17.7	17.7	18.9	37.5
53	24.8	23.0	19.1	17.9	20.5	31.5	26.3	19.4	19.9	21.5	40.2
54	27.4	25.8	21.3	21.1	23.6	34.1	28.8	21.7	21.9	24.2	43.0
55	30.5	29.0	23.4	24.8	27.2	36.8	31.8	24.1	24.7	27.0	46.5
56	33.8	32.0	26.1	27.8	30.5	39.7	35.3	27.4	28.1	30.5	50.0
57	37.1	35.1	29.6	31.2	33.7	42.5	38.8	31.1	30.8	34.7	53.5
58	40.4	38.3	33.7	34.5	36.9	45.1	42.2	34.0	33.5	38.6	57.2
59	43.8	41.8	37.6	37.1	40.8	48.6	45.6	37.5	36.5	41.6	61.1
60	47.5	45.8	41.0	40.1	44.8	53.4	48.9	42.1	39.3	45.4	63.9
61	51.0	49.8	44.5	43.5	49.3	57.6	52.0	46.3	42.7	48.7	66.1
62	54.9	54.3	50.6	47.1	54.2	61.5	55.5	49.3	46.8	52.3	69.1
63	58.8	58.4	57.2	50.1	58.3	65.3	59.1	52.5	50.6	56.1	72.6
64	62.4	62.1	60.6	52.9	61.9	69.8	62.7	55.9	54.2	60.2	75.5
65	66.2	66.2	64.5	57.4	65.7	73.9	66.3	59.0	58.8	64.6	77.6
66	70.6	70.8	70.2	63.4	70.0	77.5	70.4	63.0	63.7	68.7	80.9
67	74.6	75.0	75.5	67.5	74.3	80.9	74.2	67.9	67.9	72.3	94.4
68	77.7	78.3	79.4	71.0	78.3	83.2	77.2	71.7	71.6	75.3	86.5
69	80.5	81.2	81.6	75.2	81.2	85.5	80.0	74.8	75.1	78.3	88.3
70	83.2	83.7	83.7	78.5	83.2	88.3	82.8	78.6	78.4	81.4	90.0
71	85.8	86.2	86.1	81.2	85.6	90.6	85.5	81.9	81.0	84.4	92.0
72	88.0	88.2	88.1	83.0	88.4	92.1	87.8	84.0	83.3	87.1	94.2
73	90.1	90.2	90.3	84.7	90.7	93.8	90.0	86.5	85.7	89.6	95.7
74	92.1	92.2	92.2	87.3	92.5	95.5	91.9	89.8	88.2	91.6	96.6
75	93.6	93.8	94.0	89.5	93.8	96.8	93.5	92.1	90.3	93.3	97.3
76	94.8	94.9	95.5	90.9	94.8	97.8	94.8	93.3	92.3	94.6	97.9
77	97.7	97.7	98.1	95.9	97.7	99.1	97.7	97.0	96.6	97.5	99.1

^a^ Percentiles indicate the rank of the subject compared to other subjects of the same age subgroups within sex.

For example, a RS-11 score of 65 in a 24-year-old man indicates a percentile rank of 66.2% in the total population and of 67.0% in a group of subjects of the same age and gender. Likewise, a RS-11 score of 65 in a 24-year-old woman corresponds to a percentile rank of 66.2% in the total population and of 59.0% in the same age and gender group.

## Discussion

The present study, including more than 5000 subjects, gives evidence that the RS-11 is a reliable and valid unidimensional self-report measure for resilience in the general population. The RS-11 was found to have good internal consistency (α =.92) and construct reliability (Ω_w_ =.92). Due to the results of confirmatory factor analysis, this scale can be assumed to be unidimensional with all items loading substantially on a latent factor of resilience. Though the findings of the TLI and CFI (TLI =.911, CFI =.929) could be seen a possible limitation of the assumed unidimensionality, we still think that this deviation is of no great practical relevance; values of TLI and CFI over .9 may still indicate an adequate model fit (e.g. [70]). Furthermore, scalar invariance of the RS-11 could be confirmed across men and women, which allows comparing latent and observed means of both subgroups. Regarding the invariance tests for several age groups, the multi-group CFA confirmed only partial invariance (without the constraint of same intercepts of items 5 and 11), which hampers the comparability of mean scores of different age groups, especially between people younger versus older than 70 years. Resilience seems to fall over the lifetime in a consistent way. Least resilience was reported from 75 years on. This might be due to less energy in this period of life. Overall, in studies that used the Resilience Scale, age effects turned up when the samples had a broader age range and they were less likely to turn up in samples of a narrower age range [14] [39].

An additional main result of this study was the standardization of the RS-11 with the provision of normative data from the general population for different age and gender groups. Given that age and gender specific comparative data were generated based on subgroups consisting of N = 141 to N = 566 subjects each, the sample sizes were sufficient to provide normative data. Resilience scores varied according to gender, similar to other recent studies [71,72], yet the effect size was small, likewise reported elsewhere [14,15].

The obtained findings could be further utilized as reference categories in community studies and health care settings [2,5]. For the communities, promotion of resilience gains more and more significant importance in terms of a healthy, well-educated population [33]. Empirical findings confirm the contribution of education on resilience, having the largest effect size in our study. Resilience now also forms a key element of the United Nations International Strategy for Disaster Reduction (UNISDR) [73]. The UNISDR definition of resilience references the idea of socio-economic status in terms of system stability. Our findings in the underlying study show medium effects of the net household income on the resilience scores. Yet the higher the income, the higher the reported resilience score was, especially for the elderly (>75 years).

Specifically, the intercorrelations of the RS-11 with the life satisfaction scale are similar to intercorrelations between these concepts in other studies suggesting further construct validity of the RS-11 [14,15]. The results of the strong association with self-esteem correspond to other recent study results on the effects of self-esteem on resilience, where self-esteem together with social support accounted for 34% of the variance in resilience [74]. Furthermore, self-esteem, mindfulness and empowerment have previously been associated with better psychological functioning and resilience processes [20]. Currie et al. (2013) postulated self-esteem as mediator for psychological well-being [75]. On the basis of the results in the present study, we conclude that resilience is a distinct construct, yet related to self-esteem in terms of an internal protective factor, besides external protective factors as for example, social support networks [76]. Future longitudinal studies on resilience and self-esteem could contribute to a possible mediating effect of self-esteem on resilience. A potential limitation of this general population study is that it is a cross-sectional study which does not allow for interpretations of causality or possible mediator effects. Further evaluations of the RS-11 are necessary to demonstrate its performance in different clinical target populations. With regard to the measurement of psychological variables, it has been much debated whether or not we should measure the general health status. In the present study, results can be interpreted only on the individual's subjective perception of resilience. Characteristics of health were defined as life satisfaction, though it can comprise other areas.

With the present study that assesses the RS-11 in a representative sample of the general population, this instrument can be assumed to have good psychometric properties and the provision of norm values allow comparing the results of further studies with age and gender specific norms of the general population.

## Supporting Information

S1 File R13_KOCALEVENT_inkl GAD. (SAV)

## References

[1] Southwick SM, Bonanno GA, Masten AS, Panter-Brick C, Yehuda R (2014) Resilience definitions, theory, and challenges: interdisciplinary perspectives. Eur J Psychotraumatol 5. 10.3402/ejpt.v5.25338 PMC4185134 25317257

[2] Luthar SS, Cicchetti D, Becker B (2000) The construct of resilience: a critical evaluation and guidelines for future work. Child Dev 71: 543–562. 10953923 10.1111/1467-8624.00164 PMC1885202

[3] Rutter M (1987) Psychosocial resilience and protective mechanisms. Am J Orthopsychiatry 57: 316–331. 3303954 10.1111/j.1939-0025.1987.tb03541.x

[4] Masten A, Powell J (2003) A resilience framework for research, policy and practice In: Luthar S, editor. Resilience and Vulnerability: Adaptation in the Context of Childhood Adversities. Cambridge/New York: Cambridge University Press.

[5] Rutter M (2013) Annual Research Review: Resilience—clinical implications. J Child Psychol Psychiatry 54: 474–487. 23017036 10.1111/j.1469-7610.2012.02615.x

[6] Mohaupt S (2009) Review article: resilience and social exclusion. Social Policy & Society 8: 63–71.

[7] Seligman ME, Steen TA, Park N, Peterson C (2005) Positive psychology progress: empirical validation of interventions. Am Psychol 60: 410–421. 16045394 10.1037/0003-066X.60.5.410

[8] Geschwind N, Peeters F, Jacobs N, Delespaul P, Derom C, Thiery E. et al (2010) Meeting risk with resilience: high daily life reward experience preserves mental health. Acta Psychiatr Scand 122: 129–138. 10.1111/j.1600-0447.2009.01525.x 20064128

[9] Wichers M, Peeters F, Geschwind N, Jacobs N, Simons CJ, Derom C et al (2010) Unveiling patterns of affective responses in daily life may improve outcome prediction in depression: a momentary assessment study. J Affect Disord 124: 191–195. 10.1016/j.jad.2009.11.010 20004977

[10] Geschwind N, Nicolson NA, Peeters F, van Os J, Barge-Schaapveld D, Wichers M. (2011) Early improvement in positive rather than negative emotion predicts remission from depression after pharmacotherapy. Eur Neuropsychopharmacol 21: 241–247. 10.1016/j.euroneuro.2010.11.004 21146375

[11] Wichers MC, Myin-Germeys I, Jacobs N, Peeters F, Kenis G, Derom C et al (2007) Evidence that moment-to-moment variation in positive emotions buffer genetic risk for depression: a momentary assessment twin study. Acta Psychiatr Scand 115: 451–457. 17498156 10.1111/j.1600-0447.2006.00924.x

[12] Beutel ME, Glaesmer H, Decker O, Fischbeck S, Brahler E (2009) Life satisfaction, distress, and resiliency across the life span of women. Menopause 16: 1132–1138. 10.1097/gme.0b013e3181a857f8 19543128

[13] Beutel ME, Glaesmer H, Wiltink J, Marian H, Brähler E (2010) Life satisfaction, anxiety, depression and resilience across the life span of men. Aging Male 13: 32–39. 10.3109/13685530903296698 19842788

[14] Schumacher J, Leppert K, Gunzelmann T, Strauß B, Brähler E (2005) Die Resilienzskala—Ein Fragebogen zur Erfassung der psychischen Widerstandsfähigkeit als Personmerkmal. Zeitschrift für Klinische Psychologie, Psychiatrie und Psychotherapie 53: 16–39.

[15] Eisenhart-Rothe A, Zenger M., Lacruz M.E., Emeny R., Baumert J., Haefner S., Ladwig K.H., (2013) Validation and development of a shorter version of the resilience scale RS-11: results from the population-based KORA-age study. BMC Psychology 1: 25 10.1186/2050-7283-1-25 25566373 PMC4270035

[16] Haase JE (2004) The adolescent resilience model as a guide to interventions. J Pediatr Oncol Nurs 21: 289–299; discussion 300–284. 15381798 10.1177/1043454204267922

[17] Rosenberg M (1965) Society and the adolescent self-image. Princeton NJ: Princeton University Press.

[18] Lundman B, Viglund K, Alex L, Jonsen E, Norberg A, Fischer RS et al (2011) Development and psychometric properties of the Inner Strength Scale. Int J Nurs Stud 48: 1266–1274. 10.1016/j.ijnurstu.2011.03.006 21474137

[19] Daigneault I, Dion J, Hebert M, McDuff P, Collin-Vezina D (2013) Psychometric properties of the child and youth resilience measure (CYRM-28) among samples of French Canadian youth. Child Abuse Negl 37: 160–171. 10.1016/j.chiabu.2012.06.004 23260113

[20] Huppert FA, So TT (2013) Flourishing Across Europe: Application of a New Conceptual Framework for Defining Well-Being. Soc Indic Res 110: 837–861. 23329863 10.1007/s11205-011-9966-7 PMC3545194

[21] Bachman Desilva M, Skalicky AM, Beard J, Cakwe M, Zhuwau T, Simon JL. (2012) Longitudinal evaluation of the psychosocial wellbeing of recent orphans compared with non-orphans in a school-attending cohort in KwaZulu-Natal, South Africa. Int J Ment Health Promot 14: 162–182. 23457424 10.1080/14623730.2012.733600 PMC3583365

[22] Holden KB, Hall SP, Robinson M, Triplett S, Babalola D, Plummer V et al (2012) Psychosocial and sociocultural correlates of depressive symptoms among diverse African American women. J Natl Med Assoc 104: 493–504. 23560351 10.1016/s0027-9684(15)30215-7 PMC3660963

[23] Rotolone C, Martin G (2012) Giving up self-injury: a comparison of everyday social and personal resources in past versus current self-injurers. Arch Suicide Res 16: 147–158. 10.1080/13811118.2012.667333 22551045

[24] White JH, Magin P, Attia J, Sturm J, Carter G, Pollak M. (2012) Trajectories of psychological distress after stroke. Ann Fam Med 10: 435–442. 10.1370/afm.1374 22966107 PMC3438211

[25] Millar SL, Donnelly M (2013) Promoting mental wellbeing: developing a theoretically and empirically sound complex intervention. J Public Health (Oxf). 10.1093/pubmed/fdt075 23912464

[26] Olowokere AE, Okanlawon FA (2013) The Effects of a School-Based Psychosocial Intervention on Resilience and Health Outcomes Among Vulnerable Children. J Sch Nurs. 10.1177/1059840513501557 23962976

[27] Lamoureux BE, Palmieri PA, Jackson AP, Hobfoll SE (2012) Child Sexual Abuse and Adulthood Interpersonal Outcomes: Examining Pathways for Intervention. Psychol Trauma 4: 605–613. 23543033 10.1037/a0026079 PMC3608141

[28] Yi-Frazier JP, Yaptangco M, Semana S, Buscaino E, Thompson V, Cochrane K et al (2013) The association of personal resilience with stress, coping, and diabetes outcomes in adolescents with type 1 diabetes: Variable- and person-focused approaches. J Health Psychol. 10.1177/1359105313509846 PMC5106185 24271691

[29] Rutter M (1990) Psychosocial resilience and protective mechanism In: Rolf J, Masten A., Cicchetti D., Neuchterlein K., Weintraub S., editor. Risk and Protective Factors in the Development of Psychopathology. New York: Cambridge University Press.

[30] Rosenberg AR, Wolfe J, Bradford MC, Shaffer ML, Yi-Frazier JP, Curtis JR et al (2013) Resilience and psychosocial outcomes in parents of children with cancer. Pediatr Blood Cancer. 10.1002/pbc.24854 PMC4066960 24249426

[31] Black C, Ford-Gilboe M (2004) Adolescent mothers: resilience, family health work and health-promoting practices. J Adv Nurs 48: 351–360. 15500529 10.1111/j.1365-2648.2004.03204.x

[32] Rutten BP, Hammels C, Geschwind N, Menne-Lothmann C, Pishva E, Schruers K et al (2013) Resilience in mental health: linking psychological and neurobiological perspectives. Acta Psychiatr Scand 128: 3–20. 10.1111/acps.12095 23488807 PMC3746114

[33] Castleden M, McKee M, Murray V, Leonardi G (2011) Resilience thinking in health protection. J Public Health (Oxf) 33: 369–377. 21471159 10.1093/pubmed/fdr027

[34] Van den Hove DL, Kenis G, Brass A, Opstelten R, Rutten BP, Bruschettini M et al (2013) Vulnerability versus resilience to prenatal stress in male and female rats; implications from gene expression profiles in the hippocampus and frontal cortex. Eur Neuropsychopharmacol 23: 1226–1246. 10.1016/j.euroneuro.2012.09.011 23199416

[35] Las Hayas C, Calvete E, Gomez del Barrio A, Beato L, Munoz P, et al (2014) Resilience Scale-25 Spanish version: validation and assessment in eating disorders. Eat Behav 15: 460–463. 10.1016/j.eatbeh.2014.06.010 25064300

[36] Lossnitzer N, Wagner E, Wild B, Frankenstein L, Rosendahl J, Padierna JA et al (2014) [Resilience in chronic heart failure]. Dtsch Med Wochenschr 139: 580–584. 10.1055/s-0034-1369862 24619714

[37] Paris J, Perlin J, Laporte L, Fitzpatrick M, DeStefano J (2014) Exploring resilience and borderline personality disorder: a qualitative study of pairs of sisters. Personal Ment Health 8: 199–208. 10.1002/pmh.1261 24700757

[38] Belcher AM, Volkow ND, Moeller FG, Ferre S (2014) Personality traits and vulnerability or resilience to substance use disorders. Trends Cogn Sci 18: 211–217. 10.1016/j.tics.2014.01.010 24612993 PMC3972619

[39] Ahern NR, Kiehl EM, Sole ML, Byers J (2006) A review of instruments measuring resilience. Issues Compr Pediatr Nurs 29: 103–125. 16772239 10.1080/01460860600677643

[40] Wagnild GM, Young HM (1993) Development and psychometric evaluation of the Resilience Scale. J Nurs Meas 1: 165–178. 7850498

[41] Wagnild G (2009) A review of the Resilience Scale. J Nurs Meas 17: 105–113. 19711709 10.1891/1061-3749.17.2.105

[42] Windle G, Bennett KM, Noyes J (2011) A methodological review of resilience measurement scales. Health Qual Life Outcomes 9: 8 10.1186/1477-7525-9-8 21294858 PMC3042897

[43] Abiola T, Udofia O (2011) Psychometric assessment of the Wagnild and Young's resilience scale in Kano, Nigeria. BMC Res Notes 4: 509 10.1186/1756-0500-4-509 22112503 PMC3261834

[44] Schuhmacher J, Leppert K, Gunzelmann T, Strauß B, Brähler E (2005) Die Resilienzskala—Ein Fragebogen zur Erfassung der psychischen Widerstandsfähigkeit als Personmerkmal. Zeitschrift für klinische Psychiatrie und Psychotherapie 53.

[45] Updegraff KA, Perez-Brena NJ, Umana-Taylor AJ, Jahromi LB, Harvey-Mendoza EC (2013) Mothers' trajectories of depressive symptoms across Mexican-origin adolescent daughters' transition to parenthood. J Fam Psychol 27: 376–386. 10.1037/a0032909 23750520 PMC3840906

[46] Masten AS, Hubbard JJ, Gest SD, Tellegen A, Garmezy N, Ramirez M. (1999) Competence in the context of adversity: pathways to resilience and maladaptation from childhood to late adolescence. Dev Psychopathol 11: 143–169. 10208360 10.1017/s0954579499001996

[47] Rutter M (1993) Resilience: some conceptual considerations. J Adolesc Health 14: 626–631, 690–626. 8130234 10.1016/1054-139x(93)90196-v

[48] Wagnild GM, Collins JA (2009) Assessing resilience. J Psychosoc Nurs Ment Health Serv 47: 28–33. 10.3928/02793695-20091103-01 20000280

[49] von Eisenhart Rothe A, Zenger M, Lacruz ME, Emeny R, Baumert J, Haefner S et al (2013) Validation and development of a shorter version of the resilience scale RS-11: results from the population-based KORA—age study. BMC Psychology. 10.1186/2050-7283-1-25 PMC4270035 25566373

[50] Lowe B, Kroenke K, Grafe K (2005) Detecting and monitoring depression with a two-item questionnaire (PHQ-2). J Psychosom Res 58: 163–171. 15820844 10.1016/j.jpsychores.2004.09.006

[51] Kocalevent RD, Hinz A, Brahler E (2013) Standardization of the depression screener patient health questionnaire (PHQ-9) in the general population. Gen Hosp Psychiatry 35: 551–555. 10.1016/j.genhosppsych.2013.04.006 23664569

[52] APA (2000) Diagnostic and Statistical Manual of Mental Disorders DSM-IV-TR (4th edition). Washington DC: American Psychiatric Press.

[53] Kroenke K, Spitzer RL, Williams JB (2003) The Patient Health Questionnaire-2: validity of a two-item depression screener. Med Care 41: 1284–1292. 14583691 10.1097/01.MLR.0000093487.78664.3C

[54] Spitzer RL, Kroenke K, Williams JB, Lowe B (2006) A brief measure for assessing generalized anxiety disorder: the GAD-7. Arch Intern Med 166: 1092–1097. 16717171 10.1001/archinte.166.10.1092

[55] Lowe B, Decker O, Muller S, Brahler E, Schellberg D, Herzog W et al (2008) Validation and standardization of the Generalized Anxiety Disorder Screener (GAD-7) in the general population. Med Care 46: 266–274. 10.1097/MLR.0b013e318160d093 18388841

[56] Ferring DF, S. H. (1996) Messung des Selbstwertgefühls: Befunde zu Reliabilität, Validität und Stabilität der Rosenberg-Skala. Diagnostica 42: 284–292.

[57] Roth M, Decker O. Herzberg P.Y., Brähler E. (2008) Dimensionality and Norms of the Rosenberg Self-esteem Scale in a German General Population Sample.. European Journal of Psychological Assessment 24: 190–197.

[58] Henrich G, Herschbach P. (2000) Questions on life satisfaction (FLZM). European Journal of Psychological Assessment 16: 150–159.

[59] Beauducel A, Herzberg PY (2006) On the performance of maximum likelihood versus means and variance adjusted weighted least squares estimation in CFA. Structural Equation Modelinig 13: 186–203.

[60] Schermelleh-Engel K, Moosbrugger H., Müller H. (2003) Evaluating the fit of structural equation models. Methods of Psychological Research 8: 23–74.

[61] Arbuckle JL (2009) AMOS 18 user's guide. Crawfordville: AMOS Development Corporation.

[62] Gregorich SE (2006) Do self-report instruments allow meaningful comparisons across diverse population groups? Medical Care 44: 78–94. 10.1097/01.mlr.0000245454.12228.8f PMC1808350 17060839

[63] Byrne B (2010) Structural equation modeling with AMOS. New York: Routledge. Taylor&Francis Group.

[64] Cheung GW, Rensvold R.B. (2002) Evaluating goodness-of-fit-indexes for testing measurement invariance. Structural equation modeling 9: 233–255.

[65] Crawford JR, Garthwaite PH, Lawrie CJ, Henry JD, MacDonald MA, Sutherland J et al (2009) A convenient method of obtaining percentile norms and accompanying interval estimates for self-report mood scales (DASS, DASS-21, HADS, PANAS, and sAD). Br J Clin Psychol 48: 163–180. 10.1348/014466508X377757 19054433

[66] Bundesamt S (2006) Mikrozensus 2005. Wiesbaden.

[67] Kessler RC, Berglund P, Demler O, Jin R, Merikangas KR, Walters EE. (2005) Lifetime prevalence and age-of-onset distributions of DSM-IV disorders in the National Comorbidity Survey Replication. Arch Gen Psychiatry 62: 593–602. 15939837 10.1001/archpsyc.62.6.593

[68] Jöreskog KG (1993) Testing structural equation models In: Bollen KA, Long JS, editors. Testing structural equation models. Newbury Park: Sage Publications.

[69] Gregorich SE (2006) Do self-report instruments allow meaningful comparisons across diverse population groups? Testing measurement invariance using the confirmatory factor analysis framework. Med Care 44: S78–94. 17060839 10.1097/01.mlr.0000245454.12228.8f PMC1808350

[70] Hu L, Bentler P (1999) Cutoff Criteria for Fit Indexes in Covariance Structure Analysis: Conventional Criteria Versus New Alternatives. Structural Equation Modelinig 6: 1–55.

[71] Liebenberg L, Ungar M., Van de Vijver F. (2012) Validation of the Child and Youth Resilience Measure-28 (CYRM-28) Among Canadian Youth. Research on Social Work Practice 22: 219–226.

[72] Merrell KW, Felver-Gant J.C., Tom K.M. (2011) Development and validation of a parent report measure for assessing social-emotional competencies of children and adolescents. J Child Fam Stud 20: 529.

[73] Nations U (2005) Report of the World Conference on Disaster Reduction. Geneva: United Nations 1–42 p.

[74] Stumblingbear-Riddle G, Romans JS (2012) Resilience among urban American Indian adolescents: exploration into the role of culture, self-esteem, subjective well-being, and social support. Am Indian Alsk Native Ment Health Res 19: 1–19. 10.5820/aian.1902.2012.1 22875470

[75] Currie CL, Wild TC, Schopflocher DP, Laing L, Veugelers P (2013) Illicit and prescription drug problems among urban Aboriginal adults in Canada: the role of traditional culture in protection and resilience. Soc Sci Med 88: 1–9. 10.1016/j.socscimed.2013.03.032 23702204

[76] Barnes M, Morris K (2007) Networks, connectedness and resilience. Social Policy & Society 6: 193–197.

